# Interdisciplinary Care Provided in a Rural Setting to Patient with Below-Knee Amputation

**DOI:** 10.7759/cureus.34700

**Published:** 2023-02-06

**Authors:** Joshua A Torres, Thomas Griffin, William D Rogenmoser

**Affiliations:** 1 Physical Medicine and Rehabilitation, Edward Via College of Osteopathic Medicine, Monroe, USA; 2 Orthotics & Prosthetics, Snell's Orthotics & Prosthetics, Alexandria, USA; 3 Physical Medicine and Rehabilitation, CHRISTUS St. Frances Cabrini Hospital, Alexandria, USA

**Keywords:** prosthetics, interdisciplinary care, physiatry, physical medicine and rehabilitation (pm&r), amputee, interdisciplinary health team

## Abstract

As a result of severe injury, limb amputation remains a pivotal procedure to preserve residual function of an injured extremity. Complications following amputation can impact successful rehabilitation. This case report aims to highlight the clinical importance of interdisciplinary care demonstrated by a 65-year-old Caucasian male below-knee amputee (BKA) who presented to an amputee clinic with complaints of right distal tibia pain. He reported that he was seen at a small rural clinic and was told he had "deterioration of his tibia". Physical exam revealed a well-healed below-knee amputation stump with tenderness to palpation of the right lateral distal residual fibula. Upon prosthetic modifications managed by our prosthetist, the patient's symptoms persisted. Further work up by Physical Medicine and Rehabilitation (PM&R) revealed a sharp edge to the distal fibula and the need for surgical revision by plastic surgery. Conditions resulting from the initial operation left this patient with factors that significantly impacted the process of restoring function to this BKA. Management of care for amputees commonly involves a variety of healthcare provider consisting of, but not limited to, primary care, physiatrists, prosthetists, plastic surgeons, and physical and occupational therapists. The aim of this case report is to illustrate how the fundamental collaboration rooted in interdisciplinary care is paramount to ensure that comprehensive care is delivered to this complex patient population that reside in rural areas.

## Introduction

Amputation remains a common management option to ensure rescue of remaining limb functionality or to ensure survival, with approximately 200,000 lower extremity procedures performed per year in the United States [1]. It has been projected that there will be a twofold increase in amputee prevalence over the next 30 years secondary to comorbid conditions, leading to 3.5 million amputees in the United States [2]. Limb loss can result from diabetic neuropathy secondary to poorly controlled diabetes mellitus, peripheral vascular disease, or trauma [3,4]. Management steps following amputation focus on providing an opportunity for the patient to utilize a prosthesis, if appropriate and desired. Additional considerations include financial barriers to prosthetics and interdisciplinary care in a rural community. Complications that may arise can be related to cardiopulmonary, musculoskeletal, or neurologic reasons, or pertain to an initial surgical intervention [5]. These complications can significantly impact the patient morbidity and rehabilitation experience, especially if additional surgical revision is needed. The level of amputation is not only determined by the ability for the distal area to heal, but also it is important to consider the patient’s comfort with the introduction of the appropriate prosthesis. The healing process can be impacted by conditions that affect wound healing such as diabetes, smoking, and poor post-amputation care [6]. The most common need for revision surgery has shown to be a suboptimal residual limb resulting from the initial amputation [7]. Poor formation of the residual amputated limb limits the patient’s ability to properly utilize a prosthesis, delays patient progress during rehabilitation, and hinders reintegration back into their activities of daily living.

A robust interdisciplinary team approach that comprises, but is not limited to, primary care, physical medicine and rehabilitation (PM&R), prosthetists, plastic surgery, psychiatrists, and physical and occupational therapists has been documented as early as 1954 [8]. This interdisciplinary connection ensures that the amputee patient can be cared for in a holistic manner with the goal of restoring the patient’s function and quality of life. Interdisciplinary care is especially important when providing care in rural areas of low socioeconomic status, where there is limited access to healthcare, transportation, medical knowledge, and continuity of care [9]. Patients from rural areas who are in a position to later seek care from larger hospital systems introduce a dilemma of insufficient retrieval of medical records, which places more emphasis on history provided by patients. Poor patient historians warrant diligent collection of available data to ensure that care gaps are properly addressed. Interdisciplinary care remains essential to address the multiple care gaps seen in amputees of rural populations, while also progressing through treatment options for the patient to regain autonomy in their social and working environments.

## Case presentation

Our patient is a 65-year-old Caucasian male below-knee-amputation (BKA) with a past medical history of coronary artery disease, hyperlipidemia, myocardial infarction, peripheral artery disease, type 2 diabetes, and gout. He presented to Mid State Orthopaedic & Sports Medicine Center in Alexandria, USA, with complaints of right distal tibia residual limb pain. His initial amputation was done in January of 2018. Available records revealed that his initial amputation was performed due to chronic ischemia secondary to a blood clot in his right foot that compromised blood supply from his right ankle distally. He reported that the pain started four years after his initial amputation with daily use of his previously prescribed prosthesis but noted that the pain had significantly worsened within the last two-three months. He described the pain to be sharp and an eight out of 10 in pain (10 being maximum pain). Following amputation, he noted that he had participated in physical therapy only two-three times week for three months due to limited available transportation. He described his physical therapy sessions to have consisted of range of movement exercises for his lower extremities, muscle strengthening, balance control while standing, and ambulating both with and without his prosthesis. He reported that he had an X-ray done in a small rural town a week ago and was told that he has “bone decay” in his right residual limb. The patient denied any recent trauma or falls. He was initially evaluated by our prosthetist who suspected a neuroma and whose goal was to address the pain by making modifications to the current prosthetic socket the patient presented with and adjusting the number of socks for volume control.

**Figure 1:**
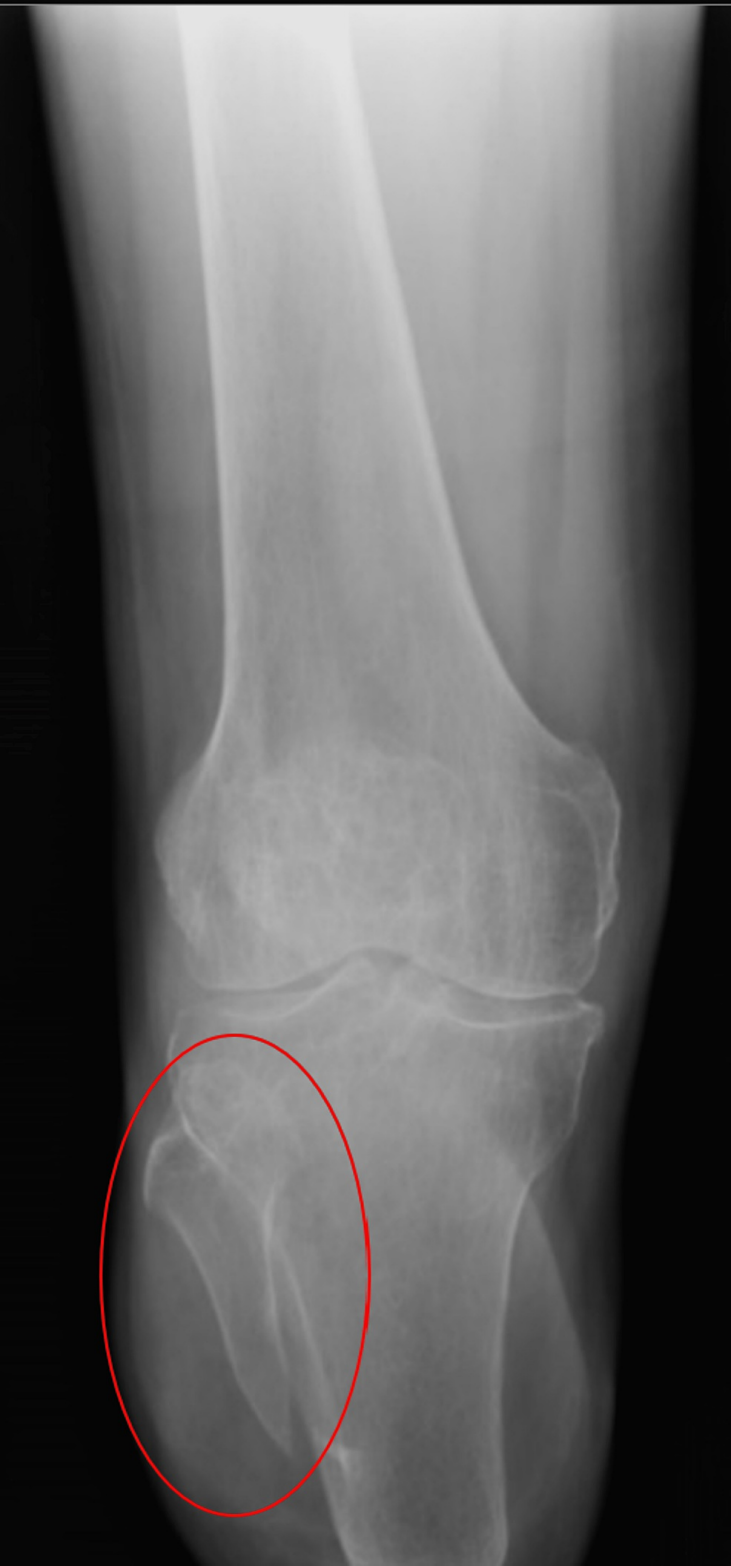
Anterior-posterior (AP) view of right tibia and fibula. The red circle marks the sharp pointed edge of the residual fibula.

**Figure 2:**
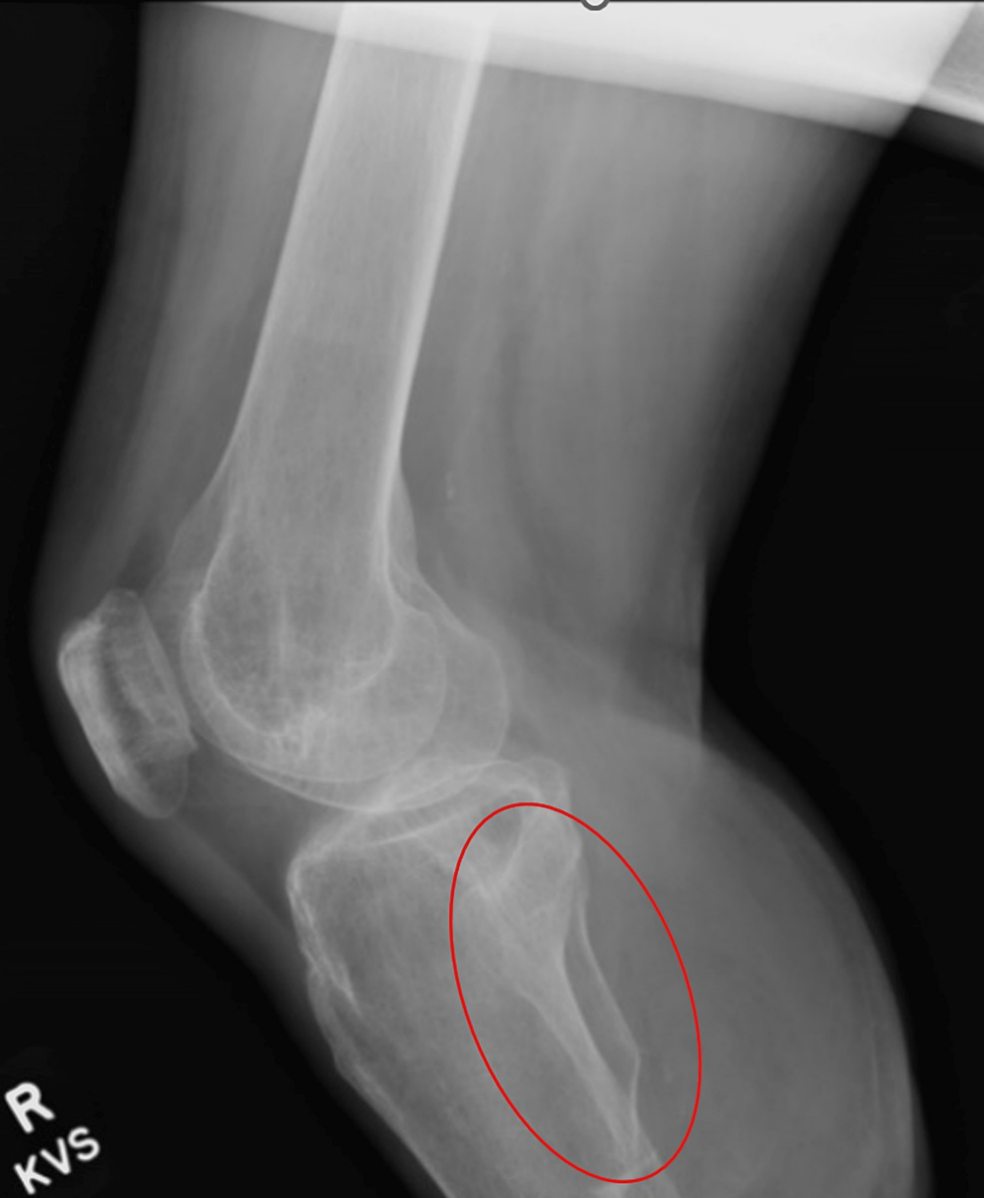
Lateral view of right tibia and fibula. The red circle marks the sharp pointed edge of the residual fibula.

Physical exam revealed a well-healed right amputation stump of a below-knee amputation. The distal limb skin revealed an invaginated scar with no erythema or signs of infection. The patient had tenderness to palpation of the right lateral distal residual fibula with full active and passive range of motion to his bilateral lower extremities. Sensation to right residual limb was intact to light touch. Normoreflexic deep tendon reflexed to his bilateral lower extremities. Motor strength was noted to be three out of five to his right quadricep muscle, revealing the ability to fully contract against gravity, and five out of five to all other extremities against resistance (five out of five noting full muscle strength against resistance). The patient showed the ability to ambulate on even and uneven surfaces at variable speeds as well as higher level activities. An anterior-posterior (AP) and lateral view x-ray of his tibia and fibula were ordered revealing a distal fibula with a pointed edge and osteophytes (Figure 1 and 2). No acute fractures were noted. Upon further evaluation by our physiatrist, the patient’s findings were found appropriate to consult plastic surgery for potential revisional surgery. Our patient was educated on the etiology for his pain and was presented options of having bone shaving and BKA revisional surgery.

Pain secondary to direct contact between the right distal fibula and prosthesis was managed by our prosthetist until surgical revision. The patient’s current prosthesis was an endoskeletal definitive prosthesis with a total surface bearing transtibial (TSB) socket with a Kinterra foot that he received from an outside provider three years ago. Prosthetic modifications were aimed towards obtaining a right TSB socket replacement with the main goal of providing extra room while also achieving a total contact fit with the proximal and distal fibula head. The socket fit was modified by using heat to mold and provide a flare to the prosthetic component in direct contact with the fibular head to relieve pressure. The patient reported reduced pain by 80% while bearing weight and walking, revealing visible improvement in his gait confidence while utilizing the modified socket. Overall, the patient displayed a dramatic improvement in selected walking speed and fastest walking speed from initial test with old socket to testing with his new socket during a 10-meter walk test. Self-selected speed improved from 0.80 m/s to 1.30 m/s. The fast gait speed improved from 1.15 m/s to 1.88 m/s. The patient had the ability and potential for ambulation with variable cadence. Overall, the new socket fit and off-loading of the initially painful right distal fibula improved upon modifications and contributed to the patient’s successful ambulation until his appointment with our plastic surgeon.

Upon presenting to our plastic surgeon during September 2022, surgical options of complete or revisional amputation were thoroughly explained and discussed. The patient-centered plan was to relieve the pain by removal of the fibular shaft remnant due to the discomfort he experienced while previously ambulating with his prosthesis. After general anesthesia was induced, the area around the fibula was injected with a dilute marcaine, lidocaine, and epinephrine mix. The patient had a 2 cm invaginated scar located on the distal end of his residual limb. It was excised as a 0.7 x 2 cm ellipse and then closed with interrupted horizontal mattress sutures to provide appropriate edge eversion. A longitudinal incision was made parallel to the long axis of the leg was made that revealed a rather brittle fibula encompassed by scar tissue. 8 cm of the distal fibula were fragmented, and removal was confirmed with intraoperative plain films to ensure that no additional bony fragments remained. The fibular head was left intact due to the amount of scarring and difficulty to dissect it while also not being the main culprit of the patient’s pain and discomfort. The fragments were submitted to pathology and revealed osteomyelitis that was cleared with doxycycline 100 mg per oral (PO) bid by his family medicine physician.

## Discussion

The common approach to addressing complications related to residual limbs starts with an interdisciplinary approach to initially identify the source of the complication. In the context of our patient, upon initial evaluation by our prosthetist, concerted clinical decision-making helped formulate a concise plan to not only address complications, but also set a goal for our patient to begin rehabilitation with other interdisciplinary providers. Goals for our interdisciplinary team included initial pain management, prosthetic fitting adjustments to reduce pain until surgical revision, and introduction to physical and occupational therapy. This outlook provides patients with amputations in rural communities with a holistic approach to address functional barriers and improving activities of daily living.

Continuity of care between disciplines during evaluation and treatment of patients can improve patient outcomes [10]. Our prosthetist consulting our physiatrist upon our patient’s initial presentation helped with fluid coordination for efficient evaluation assessment, allowing effective collection of pertinent information that related to the patient’s prior baseline activity, physical activity, and common hobbies. Our prosthetist and physiatrist also displayed their expertise when determining if our patient was fit for a prosthesis, emphasizing the importance of stability, ability to don or doff the prosthesis, and overall safety during prosthetic utilization. Upon addressing concerns, reevaluation was performed to ensure our patient felt safe and comfortable with the management plan.

A big priority for our patient was how to proceed with resuming his activities of daily living. Upon completion of our patient’s revisional surgery, initiation of physical and occupational therapy would assist with his main goal of reestablishing independence in his life that related to self-care, leisure, and outdoor activities [11]. Our physical and occupation therapists assist immensely in the status updates and progress of our amputee patient by being vigilant of any new presenting complications such as residual limb pain, phantom limb pain, overuse, and musculoskeletal issues. Recreational therapists also assist in establishing successful approaches to reintegrate patients into their activities of daily living [6].

With patient-centered care, focus on our patient’s point of view with each intervention along the process remains a top priority. Communication across each disciplinary provider when making critical decisions for interventions was essential to ensure correct steps were made toward pain relief for our patient. From our patient’s viewpoint, it was communicated that surgery can be deterring when considering additional time spent preparing for the revisional surgery, potential surgical complications, and the process of undergoing rehabilitation again. Relearning proper prosthetic utilization can include additional prosthetic fittings, relearning how to ambulate with a new fitting prosthesis and potentially the inability to use a prosthesis. From the patient’s viewpoint, there were many considerations that lay before agreeing to undergo additional surgery for pain relief. If our patient’s autonomous decision was to decline revisional surgery, our interdisciplinary team would address obstacles to ensure improvement of our patient’s quality of life.

## Conclusions

Rehabilitation involves reestablishing function and quality of life via therapy and training. This case report recalls the importance of an interdisciplinary approach when complications arise in a BKA from a rural setting. Although the path of rehabilitation is not always straightforward, there are many moving components when providing holistic care to amputee patients. Successful improvement of quality of life in this patient was demonstrated by the teamwork of clinicians from multiple disciplines to ensure that the patient can resume successful integration into daily living. It remains crucial to integrate interdisciplinary care when working with patients with amputations to ensure that they are the center of care.
